# Whole-Genome Signatures of Selection in Sport Horses Revealed Selection Footprints Related to Musculoskeletal System Development Processes

**DOI:** 10.3390/ani10010053

**Published:** 2019-12-26

**Authors:** Siavash Salek Ardestani, Mehdi Aminafshar, Mohammad Bagher Zandi Baghche Maryam, Mohammad Hossein Banabazi, Mehdi Sargolzaei, Younes Miar

**Affiliations:** 1 Department of Animal Science, Science and Research Branch, Islamic Azad University, Tehran 1477893855, Iran; Siavash.SalekArdestani@dal.ca (S.S.A.); aminafshar@gmail.com (M.A.); 2 Department of Animal Science, University of Zanjan, Zanjan 4537138791, Iran; mbzandi@znu.ac.ir; 3 Department of Biotechnology, Animal Science Research Institute of Iran (ASRI), Agricultural Research, Education & Extension Organization (AREEO), Karaj 3146618361, Iran; hossein.banabazi@gmail.com; 4 Department of Pathobiology, Veterinary College, University of Guelph, Guelph, ON NIG2W1, Canada; msargol@uoguelph.ca; 5 Select Sires Inc., Plain City, OH 43064, USA; 6 Department of Animal Science and Aquaculture, Dalhousie University, Truro, NS B2N5E3, Canada

**Keywords:** horse, musculoskeletal, non-sport, signature of selection, sport

## Abstract

**Simple Summary:**

Throughout horse industry modernization, sport horse breeds have been genetically evolved in accordance to their abilities in sport disciplines providing an opportunity to study selection signatures in the genome level. Future selection strategies of sport horse breeds can be optimized by improving our knowledge of genomic signatures of selection. The main goals of this study are identifying and investigating the genes and their biological pathways underlying selective pressures in sport and non-sport horse breeds. Here, we detected 49 genes as selective signals using fixation index, nucleotide diversity and Tajima’s D approaches. Intriguingly, our findings in functional enrichment analysis revealed the selection footprints related to musculoskeletal system development. Detected candidate genes and biological pathways in this study may be helpful to widen our perspective in recent breeding efforts and genomic evolutionary mechanisms in sport horse breeds.

**Abstract:**

Selective breeding has led to gradual changes at the genome level of horses. Deciphering selective pressure patterns is progressive to understand how breeding strategies have shaped the sport horse genome; although, little is known about the genomic regions under selective pressures in sport horse breeds. The major goal of this study was to shed light on genomic regions and biological pathways under selective pressures in sport horses. In this study, whole-genome sequences of 16 modern sport and 35 non-sport horses were used to investigate the genomic selective signals of sport performance, by employing fixation index, nucleotide diversity, and Tajima’s D approaches. A total number of 49 shared genes were identified using these approaches. The functional enrichment analysis for candidate genes revealed novel significant biological processes related to musculoskeletal system development, such as limb development and morphogenesis, having been targeted by selection in sport breeds.

## 1. Introduction

The livestock species have been shaped by humans according to their needs and purposes since the beginning of domestication process. The establishment of studbooks and modernization of breeding methods have played key roles to increase the selective pressure for traits of interest in livestock species [1]. In order to improve athletic performance of horses, the selection programs based on modern methods started in the late 20th century, particularly in Europe, by warmblood horse breeding organizations [2]. Mainly, the German and Dutch warmbloods have been bred as sport breeds and used for three major athletic disciplines: dressage, showjumping, and eventing [3].

Swift technical advances of genome sequencing have made the large-scale sequencing data available for investigation of candidate genes associated with economic traits [4,5,6]. Particularly, revealing the genetic architecture of the horse athletic performance is crucial for breeding organizations to optimize the selection and mating strategies that directly affects the marketability of sport horse breeds [7]. Although the study of equine genomics started late in comparison with the genomics studies of other species [6], it has been improved increasingly in recent years [3,8,9,10,11]. The identification of genomic regions that have been subjected to selective pressure as signatures of selection is one of the approaches to screen the candidate genes for economic traits in horses [3,10] and other livestock species [5,12,13]. Detecting the candidate genes for traits such as reproduction [14], racing performance [15], body size [10,16,17], and type [18] was the main objective in recent horse signatures of selection studies. The main goals of this study are to investigate the population genetic structure, verify the role of effective genes, and detect novel candidate genes associated with athletic performance, using whole-genome sequences of sport and non-sport horse breeds. Tracing the footprints of selection in the equine genome may help us better understand the selection role for athletic disciplines specifically in sport breeds during the evolution. Additionally, it can be useful to optimize the single nucleotide polymorphism (SNP) arrays that are widely used in breeding programs based on genomic evaluation.

## 2. Materials and Methods

### 2.1. Animals

The whole-genome sequence data (Supplementary Table S1) of Baden-Wurttemberg (n = 1), Dutch warmblood (n = 1), Hanoverian (n = 6), Holstein (n = 2), Oldenburg (n = 3), Trakehner (n = 1), and Westphalian (n = 2) as seven sport breeds, as well as, Akhal-Teke (n = 3), American Miniature (n = 2), Arabian (n = 2), Connemara pony (n = 4), Dülmen pony (n = 1), Friesian (n = 1), Jeju pony (n = 2), Noriker (n = 1), Percheron (n = 1), Saxon-Thuringian Heavy Warmblood (n = 1), Shetland pony (n = 4), Sorraia (n = 1), Standardbred (n = 6), Thoroughbred (n = 5), and Welsh pony (n = 1) as 15 non-sport breeds, were downloaded from the European Nucleotide Archive (https://www.ebi.ac.uk/ena). Former studies were used to determine the sport and non-sport breeds [2,3].

### 2.2. Whole-Genome Mapping and Variant Calling

For each raw whole-genome sequence, the quality control was performed by FastQC (version 0.11.6, http://www.bioinformatics.babraham.ac.uk/projects/fastqc), after converting their format (SRA to fastq format). The low-quality bases and adaptors were filtered by Trimmomatic 0.36 [19]. The qualified reads were aligned against the reference horse genome (EquCab2.0, ftp://ftp.ensembl.org/pub/release-93/fasta/equus_caballus/dna), using Burrows–Wheeler Aligner 0.7.17-r1188 (http://bio-bwa.sourceforge.net) [20]. The PCR duplicates were detected and removed using Picard 2.17.11 (https://broadinstitute.github.io/picard). Recalibrating based quality score (by applying “BaseRecalibrator” and “BQRS” arguments), detecting SNPs, and insertion/deletion (by applying “HaplotypeCaller” argument) were conducted using suggested workflow in Genome Analysis Toolkit 3.8 [21]. After discarding X chromosomes and insertion/deletion (in all chromosomes), in order to detect high-quality SNPs, all SNPs exhibiting mapping quality <25, quality by depth <2, genotype quality <40, and fisher strand >60 and mapping quality rank sum <−12.5, minor allelic frequency <0.01, Hardy–Weinberg *p*-value < 0.001, and genotype frequency <0.1, as well as individuals with more than 10% missing genotypes, were marked and removed.

### 2.3. Population Genetic Structure

The neighbor-joining phylogenetic analysis was performed using VCF-kit 0.1.6 (https://vcf-kit.readthedocs.io/en/latest) [22] and FigTree 1.4.3 (http://tree.bio.ed.ac.uk/software/figtree), to assess the genetic distance among all individuals. To have an overview of the population structure for under-studied individuals and breeds, principal component analysis (PCA) and Bayesian model-based approach were carried out, using PLINK 1.9 (https://www.cog-genomics.org/plink) [23] and ADMIXTURE 1.3 (http://software.genetics.ucla.edu/admixture) [24] software, respectively. After PCA visualization, a clustering approach was performed on PCA results, using k-mean clustering algorithm in R software (https://cran.r-project.org). In addition, long runs of homozygote regions for sport and non-sport groups were quantified using PLINK 1.9 software by homozyg command and default options follows: homozyg-window-snp 50, homozyg-window-het 1, homozyg-window-missing 5, homozyg-gap 1000, homozyg-density 50, homozyg-snp 100, and homozyg-kb 1000 [23]. Furthermore, linkage disequilibrium (LD) was estimated and visualized using PopLDdecay 1.01 software (https://github.com/BGI-shenzhen/PopLDdecay) and a perl script, respectively [25].

### 2.4. Genome-Wide Selective Signals Scan and Gene Ontology (GO)

Here, two main approaches including fixation index (F_st_) [26] and pairwise nucleotide diversity (θπ) [27] were used to detect the signatures of selection differentiating sport breeds from non-sport breeds. To identify more reliable selective signal regions, the Tajima’s D values [28] in sport group were calculated for shared selective signal windows between F_st_ and θπ methods. A sliding window approach (100 kb with a step size of 50 kb) was used to calculate F_st_ and θπ using VCFtools 0.1.15 (http://vcftools.sourceforge.net/index.html) [29], and Tajima’s D values using VCF-kit 0.1.6 [22] software. After performing Z transformation of F_st_ (Z(F_st_)) values using “scale” command in R software, the shared windows in top 1% of Z(F_st_) and $\log_{2}\left(\frac{{\theta \pi }_{\left(\mathrm{Non}-\mathrm{sport}\right)}}{{\theta \pi }_{\left(\mathrm{Sport}\right)}}\right)$ values were validated using Tajima’s D values in sport group. Also, by employing “ranges” function, a custom-made script was applied in R software, to extract overlapped regions between long homozygous and genomic selection signature regions (shared regions between top 1% of Z(F_st_), top 1% of $\log_{2}\left(\frac{{\theta \pi }_{\left(\mathrm{Non}-\mathrm{sport}\right)}}{{\theta \pi }_{\left(\mathrm{Sport}\right)}}\right)$, and Tajima’s D).

Gene ontology analysis was performed using Gene Ontology Consortium (http://geneontology.org), to investigate the biological enrichment of genes under selective pressure.

## 3. Results and Discussion

### 3.1. Genomic Variants and Population Genetic Structure

The high-quality paired-end reads of 51 sport and non-sport horses obtained from NextSeq 500, Illumina MiSeq and HiSeq (2000, 2500, and 3000) platforms were aligned to equine genome reference (94.59%–99.84%) with 14.42X average coverage (Supplementary Table S1). These data yielded 14,843,096 high-quality SNPs after variant calling and quality control steps.

The population genetic structure studies have been effective to describe the impact of evolutionary processes such as biogeographic history and selection, and they are also spotlights to determine the genetic variation among populations [30]. Former studies have revealed that combining the results of different population genetic structure analyses such as PCA, phylogenetic, and Bayesian approaches can be helpful to provide a comprehensive interpretation for genetic variation in livestock populations e.g., horse [6,10], goat [5], and sheep [4]. Here, we utilized the abovementioned methods to unfold population genetic structure of the studied horse breeds.

**Figure 1:**
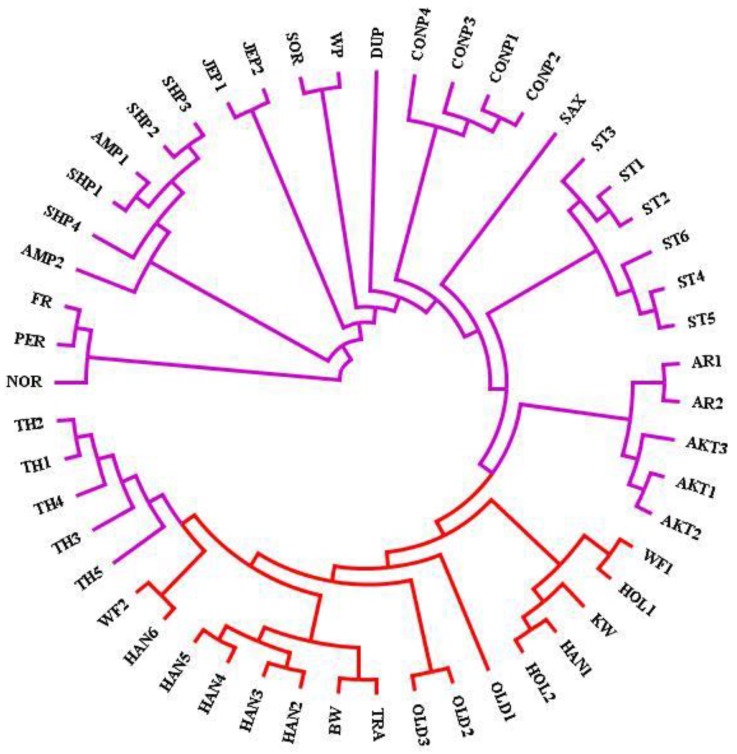
Neighbor-joining phylogenetic tree for sport and non-sport horse breeds. The sport breeds (red lines) are Baden-Wurttemberg (BW), Dutch warmblood (KW), Hanoverian (HAN), Holstein (HOL), Oldenburg (OLD), Trakehner (TR), and Westphalian (WF). Non-sport breeds (purple lines) are Akhal-Teke (AKT), American Miniature (AMP), Arabian (AR), Connemara pony (CONP), Dülmen pony (DUP), Friesian (FR), Jeju pony (JEP), Noriker (NOR), Percheron (PER), Saxon-Thuringian Heavy Warmblood (SAX), Shetland pony (SHP), Sorraia (SOR), Standardbred (ST), Thoroughbred (TH), and Welsh pony (WP).

In this study, the phylogenetic analysis illustrated sport breeds including Dutch warmblood (KW), Baden–Wurttemberg (BW), Hanoverians (HAN), Holsteins (HOL), Oldenburgs (OLD), Trakehner (TRA), and Westphalians (WF) in a main branch (Figure 1, red color). Similar to former studies, there was a close genetic relationship between sport horses and Thoroughbred [10,31,32]. Arabians and Akhal-Tekes, which were Middle Eastern horse breeds, were classified in one branch, similar to Petersen et al. [16] and Kader et al. [10]; this might be due to their shared biogeographic history and founder lines [10,16]. Standardbreds, Connemara ponies and Jeju ponies were properly grouped in unique separated branches. Saxon-Thuringian Heavy Warmblood was placed between Standardbred and Connemara pony branches in the phylogenetic tree. To the best of our knowledge, this is the first report of Saxon-Thuringian Heavy Warmblood phylogenetic analysis showing its close genetic relationship with these two breeds. The phylogenetic analysis demonstrated the close genetic relationship among Connemara ponies, Dülmen pony, Sorraia, and Welsh pony. The close genetic relationships between Dülmen pony and Sorraia had also been observed in a previous study using PCA analysis [33]. As expected, Shetland and American Miniatures were classified in one branch because of their common ancestors [34]. Percheron, Noriker and Friesian were categorized in a branch that is similar to the close genetic distance between Noriker and Friesian, confirmed by a previous study based on mitochondrial-DNA data [35].

**Figure 2:**
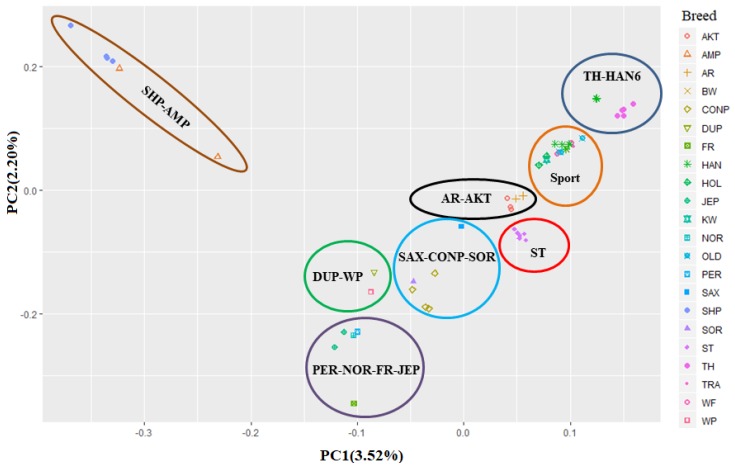
Principle component analysis for sport and non-sport horse breeds. The sport breeds are Baden-Wurttemberg (BW), Dutch warmblood (KW), Hanoverian (HAN), Holstein (HOL), Oldenburg (OLD), Trakehner (TR), and Westphalian (WF). Non-sport breeds are Akhal-Teke (AKT), American Miniature (AMP), Arabian (AR), Connemara pony (CONP), Dülmen pony (DUP), Friesian (FR), Jeju pony (JEP), Noriker (NOR), Percheron (PER), Saxon-Thuringian Heavy Warmblood (SAX), Shetland pony (SHP), Sorraia (SOR), Standardbred (ST), Thoroughbred (TH), and Welsh pony (WP). The PC1 and PC2 are the first two principal components.

In the individual-scaled PCA analysis, 3.52% and 2.20% of the genetic variation were explained by the first two principal components, respectively (Figure 2). In contrast to the phylogenetic analysis, Percheron, Noriker, Friesian, and Jeju ponies (PER–NOR–FR–JEP group) were classified in a cluster by PCA analysis. Saxon-Thuringian Heavy Warmblood and Connemara ponies along with Sorraia (SAX–CONP–SOR group), as well as, Dülmen pony and Welsh pony together (DUP–WP), were grouped in separate clusters. Except for HAN6, all sport individuals were classified in one group (Sport). Thoroughbreds and HAN6 were grouped in a shared cluster (TH–HAN6), probably due to hybridization between these breeds. This close genetic relationship was also confirmed by our phylogenetic analysis. Other clusters (AR–AKT, ST, and SHP–AMP) in the PCA analysis supported the phylogenetic results.

**Figure 3:**
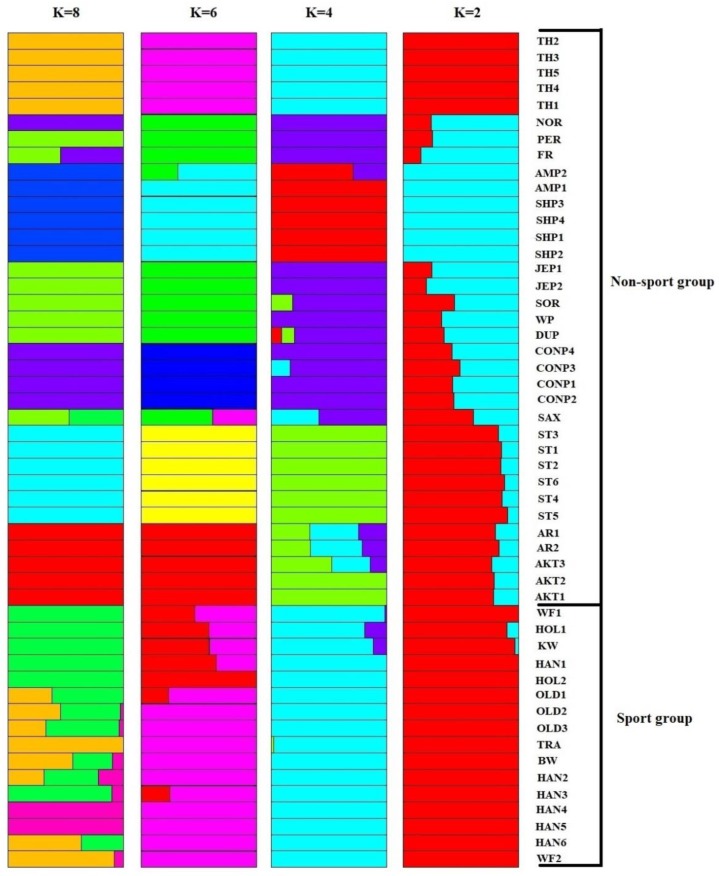
Bayesian clustering plot for 4 K values (K = 2, K = 4, K = 6, and K = 8) in 51 horses. Each horse is indicated horizontally which is divided into colored blocks and each color demonstrates one ancestral population. The sport breeds are Baden-Wurttemberg (BW), Dutch warmblood (KW), Hanoverian (HAN), Holstein (HOL), Oldenburg (OLD), Trakehner (TR), and Westphalian (WF). Non-sport breeds are Akhal-Teke (AKT), American Miniature (AMP), Arabian (AR), Connemara pony (CONP), Dülmen pony (DUP), Friesian (FR), Jeju pony (JEP), Noriker (NOR), Percheron (PER), Saxon-Thuringian Heavy Warmblood (SAX), Shetland pony (SHP), Sorraia (SOR), Standardbred (ST), Thoroughbred (TH), and Welsh pony (WP).

When K = 2 in the whole-genome admixture clustering based on the Bayesian approach, all of horses were categorized into four main groups (Figure 3). These groups include the following: (1) Thoroughbreds and sport breeds; (2) Noriker, Saxon-Thuringian Heavy Warmblood, Percheron, Friesian, Sorraia, Dülmen pony, Connemara ponies, Welsh pony, and Jeju ponies; (3) American Miniature and Shetland ponies; and (4) Standardbreds, Arabians, and Akhal-Tekes. The Standardbreds at K = 6 and K = 8, American Miniature and Shetland ponies at K = 8 and K = 2, Arabians and Akhal-Tekes at K = 6 and K = 8, and Connemara ponies at K = 6 were clustered as unique groups, which were also supported by our phylogenetic tree. It should be noted that in aforementioned analyses, the interpretation of the results related to the breeds with one individual such as Baden-Wurttemberg, Dutch warmblood, Trakehner, Dülmen pony, Friesian, Noriker, Percheron, Saxon-Thuringian Heavy Warmblood, Sorraia, and Welsh pony requires further investigation using larger sample size.

**Figure 4:**
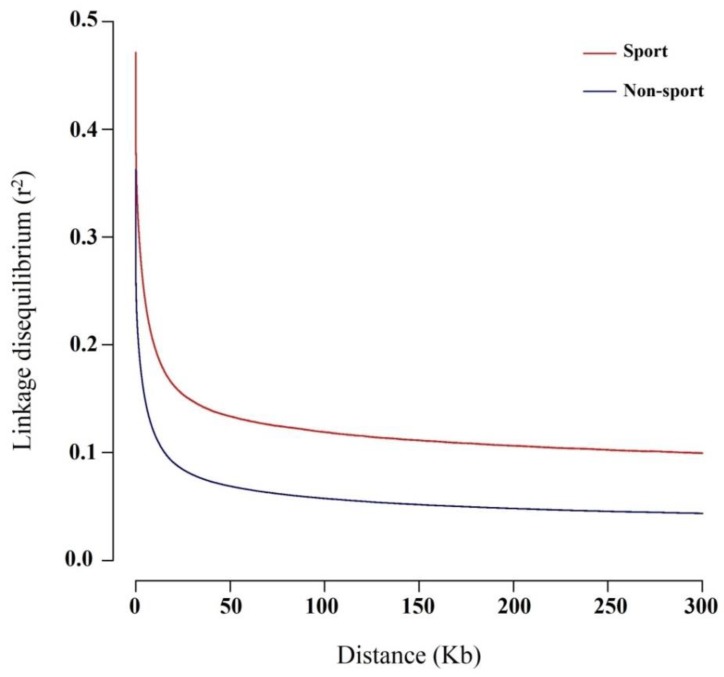
Linkage disequilibrium decay among sport (red) and non-sport (blue) groups.

LD patterns are affected by a range of demographic force and evolutionary trend [36]; therefore, investigation of LD patterns can be informative in population demography [6]. The LD patterns between sport and non-sport groups indicated that the mean of r^2^ in both groups dropped rapidly at approximately 10 Kb (Figure 4). The means of r^2^ at 300 Kb for sport and non-sport groups were 0.09 and 0.04, respectively. In a previous study, the mean of r^2^ at 300 Kb was approximately 0.08 for Hanoverian as a sport breed, which is in agreement with our results [32]. 

Size and frequencies of long contiguous segments of homozygous genotypes in the genome level known as runs of homozygosity (ROH) are valuable for detection of genetic connectedness between and within populations, as well as, recent inbreeding [6,14]. Additionally, identifying the ROHs can be helpful to detect selective signals [14] and mutations related to recessive diseases in human [37]. In this study, we quantified ROHs for each individual to assess the recent inbreeding and genetic connectedness among individuals (Supplementary Table S2). The total number of ROHs for sport and non-sport breeds were 820 and 2400, respectively. The Supplementary Figure S1 indicates the percentage of ROHs that are distributed in different lengths, in which the highest frequencies were detected for 1–1.5 Mb in both sport and non-sport horse breeds. Frequency patterns and the extent of ROHs depend on the population size, ancestry of animals, and recent or ancient selection pressures [38]. The most enriched ROHs chromosome (ECA1) had 8.65% of ROHs in the sport group and 10.79% of ROHs in the non-sport group (Supplementary Figure S2), which might be due to the fact that this chromosome is the largest chromosome in horse. The highest length-size of ROHs in the sport and non-sport groups were located at ECA21: 13.12–20.41 Mb (average SNP density = 0.184) and ECA15: 37.74–43.72 Mb (average SNP density = 0.211) in DUP and HAN3, respectively (Supplementary Table S2).

### 3.2. Selective Signals Detection

The performance quality of sport breeds (e.g., show-jumping competitions) depends on various factors such as muscular power and balance [39]. A few candidate genes related to sport performance have been identified by previous genome-wide association studies [7]. However, assuming the sport performance as a simple trait that is controlled by a few genes can be unrealistic [7], and, thus, signatures of selection studies may identify novel candidate genes related to this complex trait. Additionally, combining the results of different signatures of selection approaches can increase the reliability, because different methods can focus on different genomic selective signals that have been subjected to selection in varied time scales [40]. Our main selection signature tests in this study were fixation index [26] and pairwise nucleotide diversity [27] based on population differentiation and allele frequency spectrum, respectively. Furthermore, we calculated the Tajima’s D values in sport group for shared selective signals between fixation index and pairwise nucleotide diversity approaches to improve the reliability and efficiency of the results.

**Figure 5:**
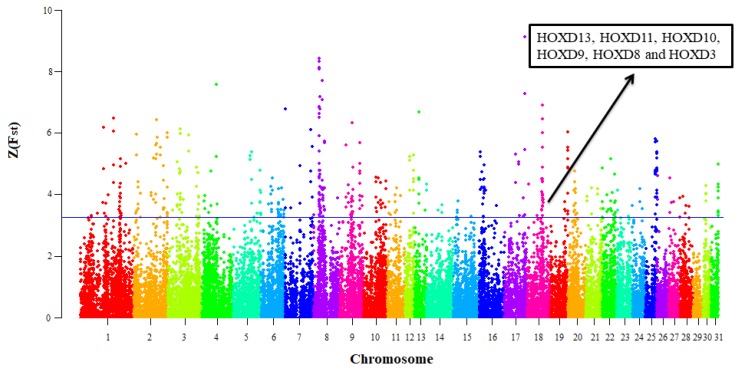
The distribution of absolute Z(F_st_) values in 31 horse autosomes. The data points above the horizontal line (blue line, Z(F_st_) ≥ 3.26) are top 1% Z(F_st_) values. The *HOXDs* located on ECA18: 54.55–54.65Mb were overrepresented genes in top 1% $\log_{2}\left(\frac{{\theta \pi }_{\left(\mathrm{Non}-\mathrm{sport}\right)}}{{\theta \pi }_{\left(\mathrm{Sport}\right)}}\right)$.

After Z-transformation of the F_st_ for each window of 100 kb with a step size of 50 kb, a total of 448 windows including 379 genes were detected as selective signals (Figure 5). The Z(F_st_) values followed the normal distribution (Supplementary Figure S3); the range of Z(F_st_) values of windows was from 3.27 to 9.13 located on ECA1: 137.75–137.85 Mb and ECA17: 71.90–72 Mb, respectively (Supplementary Table S3). The ECA17: 71.90–72 Mb is an intragenic region. Furthermore, we identified several candidates as selective signals including *LCORL* and *NCAPG* as wither height regulators [8], and also, *MYO5C* that had been detected as a selective signal for muscular function in four German warmblood populations in a former study [3].

**Figure 6:**
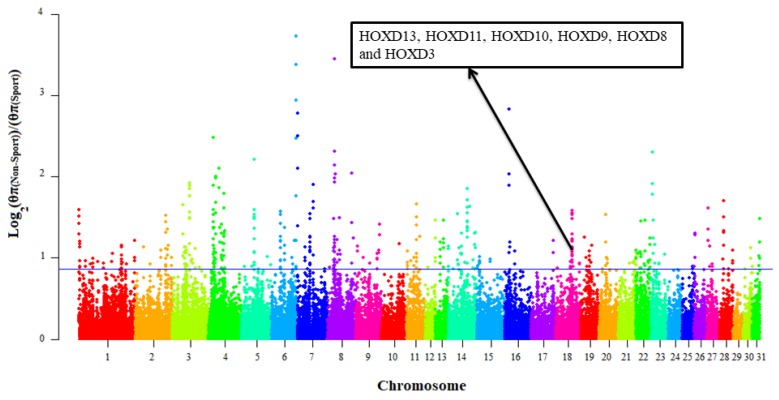
The $\log_{2}\left(\frac{{\theta \pi }_{\left(\mathrm{Non}-\mathrm{sport}\right)}}{{\theta \pi }_{\left(\mathrm{Sport}\right)}}\right)$ ratios distribution in 31 horse autosomes. The data points above the horizontal line (blue line, $\log_{2}\left(\frac{{\theta \pi }_{\left(\mathrm{Non}-\mathrm{sport}\right)}}{{\theta \pi }_{\left(\mathrm{Sport}\right)}}\right)$ ≥ 0.86) are top 1% $\log_{2}\left(\frac{{\theta \pi }_{\left(\mathrm{Non}-\mathrm{sport}\right)}}{{\theta \pi }_{\left(\mathrm{Sport}\right)}}\right)$ ratios. The *HOXDs* located on the ECA18: 54.55–54.65 Mb were overrepresented genes in the top 1% Z(F_st_) values.

The transformed pairwise nucleotide diversity ratios ($\log_{2}\left(\frac{{\theta \pi }_{\left(\mathrm{Non}-\mathrm{sport}\right)}}{{\theta \pi }_{\left(\mathrm{Sport}\right)}}\right)$) were calculated in windows similar to that of F_st(Sport-Non-sport)_ approach (Figure 6). The $\log_{2}\left(\frac{{\theta \pi }_{\left(\mathrm{Non}-\mathrm{sport}\right)}}{{\theta \pi }_{\left(\mathrm{Sport}\right)}}\right)$ ratios followed normal distribution (Supplementary Figure S3). In the top 1% of $\log_{2}\left(\frac{{\theta \pi }_{\left(\mathrm{Non}-\mathrm{sport}\right)}}{{\theta \pi }_{\left(\mathrm{Sport}\right)}}\right)$ ratios, a total number of 448 windows including 388 genes were identified as selection signatures (Supplementary Table S3). The highest $\log_{2}\left(\frac{{\theta \pi }_{\left(\mathrm{Non}-\mathrm{sport}\right)}}{{\theta \pi }_{\left(\mathrm{Sport}\right)}}\right)$ ratio was observed for a window located on ECA6: 81.35–81.45Mb containing a novel gene (ENSECAG00000026823). The ortholog of this novel gene is *HMGA2* in human and duck that is related to body height [8,41] and worth further investigation in equine genome. Evidently, the wither height has been subjected as an economic trait by sport horse breeding associations such as Royal Dutch Sport Horse (https://www.kwpn.org/). A signatures of selection study on sport horse breeds revealed several candidate genes related to wither height [3].

**Figure 7:**
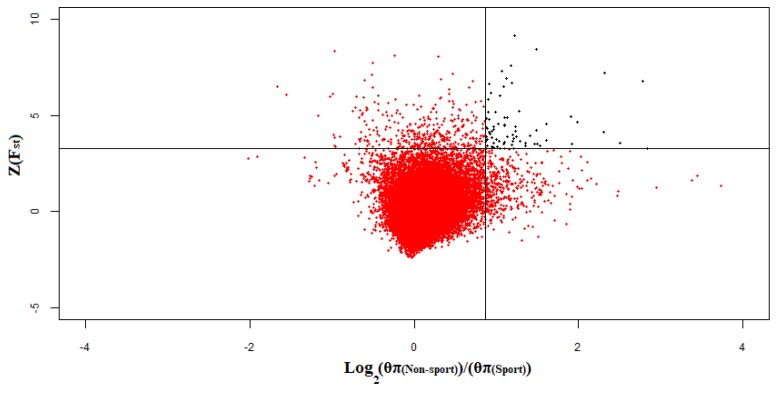
Shared windows of the top 1% $\log_{2}\left(\frac{{\theta \pi }_{\left(\mathrm{Non}-\mathrm{sport}\right)}}{{\theta \pi }_{\left(\mathrm{Sport}\right)}}\right)$ and Z)F_st_). Data points located on the right side of the vertical line (top 1% $\log_{2}\left(\frac{{\theta \pi }_{\left(\mathrm{Non}-\mathrm{sport}\right)}}{{\theta \pi }_{\left(\mathrm{Sport}\right)}}\right)$ ratios, where $\log_{2}\left(\frac{{\theta \pi }_{\left(\mathrm{Non}-\mathrm{sport}\right)}}{{\theta \pi }_{\left(\mathrm{Sport}\right)}}\right)$ ratio is 0.86), and above horizontal line (top 1% Z(F_st_), where Z(F_st_) is 3.26), are identified as shared selective genomic regions.

**Figure 8:**
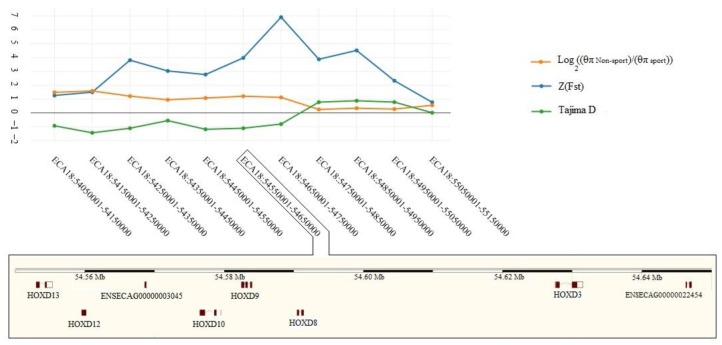
The $\log_{2}\left(\frac{{\theta \pi }_{\left(\mathrm{Non}-\mathrm{sport}\right)}}{{\theta \pi }_{\left(\mathrm{Sport}\right)}}\right)$ ratios, Z(F_st_) and Tajima’s D values distributions for each 100 kb window in ECA18: 54.05–55.15Mb. The ECA18: 54.55–54.65 Mb window includes HOXDs that are related to musculoskeletal system development processes.

The shared windows (n = 69) between F_st(Sport–Non-sport)_ and $\log_{2}\left(\frac{{\theta \pi }_{\left(\mathrm{Non}-\mathrm{sport}\right)}}{{\theta \pi }_{\left(\mathrm{Sport}\right)}}\right)$ in the top 1% consisting 65 genes were selected as selective signals (Figure 7). Finally, after discarding windows with Tajima’s D values >0, a total number of 51 windows including 49 genes remained as genomic selective signal regions (Supplementary Table S3). Although, two former studies have revealed genomic selection signature regions using ROH approach in horse [14,42], there was no overlapped region between ROHs and detected genomic selective signal regions in our study, that might be due to discarding some genomic selective signal regions by employing three approaches, various breeds, and small sample size. The lowest Tajima’s D value (–2.82) was observed in a window located on ECA7: 0.15–0.25Mb. This region contained *ARHGAP45*, *POLR2E* and *SBNO2* genes. Additionally, *HOXD* gene cluster including *HOXD13*, *HOXD11*, *HOXD10*, *HOXD9*, *HOXD8* and *HOXD3* located on ECA18 was detected by all three approaches (Figure 8). In Arabian horses, a deletion of 2.7 Kb near to *HOXD3* is related to occipitoatlantoaxial malformation as craniocervical junction abnormality [43]. This phenotype is closely related to poll angle and neck posture [44]. The attachments of the head and neck play pivotal roles in athletic ability, movement, flexion, and balance of horses [45]. Furthermore, successes in dressage performance depend on perfect horse balance and locomotion maneuverability [46]. The B cluster of *HOX* gene was detected as a selective signal region in sport German warmblood populations [3].

A biological enrichment analysis for 49 genes under positive selective pressure revealed several GO categories (Supplementary Figure S4) associated with cellular component organization or biogenesis (GO:0071840), cellular process (GO:0009987), localization (GO:0051179), reproduction (GO:0000003), biological regulation (GO:0065007), response to stimulus (GO:0050896), developmental process (GO:0032502), multicellular organismal process (GO:0032501), metabolic process (GO:0008152), and immune system process (GO:0002376). Significant biological processes under selective pressure in sport breeds (Table 1) were the appendage development (GO:0048736), appendage morphogenesis (GO:0035107), embryonic appendage morphogenesis (GO:0035113), embryonic morphogenesis (GO:0048598), pattern specification process (GO:0007389), skeletal system development (GO:0001501), forelimb morphogenesis (GO:0035136), limb development (GO:0060173), limb morphogenesis (GO:0035108), and embryonic limb morphogenesis (GO:0030326). Intriguingly, *HOXD9* and *HOXD10* genes are related to all of these biological processes. The *HOX* genes have several clusters such as A, B, C, and D [47], and they play key roles in the axial and appendicular skeleton development. The limb skeleton along the proximodistal axis is patterned by the paralogs of *HOX9*, *HOX10*, *HOX11*, *HOX12*, and *HOX13* [48]. Recently, the effective determinant roles of *HOX9* paralogs have been defined in patterning anteroposterior axis of the forelimb [49]. The morphological variation associated with hind limb integumentary appendages in mammals results from adaptive development in evolution trend [50]. Considering our significant results in biological enrichment analysis, the GOs related to limb morphogenesis and development process are highlights of our findings. Regarding the management of selection strategies in sport horse breeding associations, the associated traits with limb such as limb health and conformation most probably have been under selective pressure. The pivotal role of limbs has been observed in the previous studies in dressage [46] and show-jumping horses [39,51]. Clearly, the genes related to performance and limb health are classified under potential candidate genes for show-jumping performance [52]. Furthermore, there is a significant genetic correlation between health of limbs and athletic performance in German warmbloods [53]. A former kinematic study revealed that the contribution of fore and hind limbs plays an important role during the take-off in horse jumping [51]. Moreover, the muscles of hind limbs generate the most amount of force in jumping [39]. The role of limbs is to create the ground reaction forces during the movement and these forces are necessary for creating balance in dressage performance [46].

## 4. Conclusions

In this study, we detected the genomic regions under selective pressure in sport horse breeds, using whole-genome comparative analyses. By using three signatures of selection methods, 49 genes were identified as selective signals that were enriched for ten significant biological processes. Intriguingly, most of these biological processes were related to important musculoskeletal system development processes, such as limb development and morphogenesis. Our findings may provide novel insights into the current selection strategies for athletic ability and shed light on evolutionary mechanisms in the genome of sport horse breeds, which can be helpful for future selection strategies of sport horse breeds. Furthermore, the identified candidate genes can be employed in optimizing the SNP arrays, which have been recently used in some sport breeding associations. 

## Figures and Tables

**Table 1:** Significant biological process under selective pressure in sport breeds.

Biological Process	Genes	FDR
appendage development (GO:0048736)	*HOXD9*, *HOXD10*, *HOXD12*, *HOXD13*, and *LNPK*	0.008
appendage morphogenesis (GO:0035107)	*HOXD9*, *HOXD10*, *HOXD12*, *HOXD13*, and *LNPK*	0.006
embryonic appendage morphogenesis (GO:0035113)	*HOXD9*, *HOXD10*, *HOXD12*, *HOXD13*, and *LNPK*	0.006
embryonic limb morphogenesis (GO:0030326)	*HOXD9*, *HOXD10*, *HOXD12*, *HOXD13*, and *LNPK*	0.011
embryonic morphogenesis (GO:0048598)	*HOXD9*, *HOXD10*, *HOXD12*, *HOXD13*, *MAFB*, *FBN1*, and *LNPK*	0.013
forelimb morphogenesis (GO:0035136)	*HOXD9*, *HOXD10*, and *LNPK*	0.032
limb development (GO:0060173)	*HOXD9*, *HOXD10*, *HOXD12*, *HOXD13*, and *LNPK*	0.01
limb morphogenesis (GO:0035108)	*HOXD9*, *HOXD10*, *HOXD12*, *HOXD13*, and *LNPK*	0.008
pattern specification process (GO:0007389)	*HOXD8*, *HOXD9*, *HOXD10*, *HOXD12*, *HOXD13*, and *MAFB*	0.015
skeletal system development (GO:0001501)	*HOXD8*, *HOXD9*, *HOXD10*, *HOXD12*, *HOXD13*, and *FBN1*	0.03

FDR: False discovery rate.

## Data Availability

The raw whole-genome sequences analyzed in the current study with their sample-accession numbers (first column in Supplementary Table S1) are available in the European Nucleotide Archive (https://www.ebi.ac.uk).

## Supplementary Materials

The following are available online at https://www.mdpi.com/2076-2615/10/1/53/s1. Figure S1: Distribution of homozygote segments in different lengths (1 < length ≤ 1.5, 1.5 < length ≤ 2, 2 < length ≤ 2.5, 2.5 < length ≤ 3, 3 < length ≤ 3.5, 3.5 < length ≤ 4, and length < 4 Mb) Figure S2: The distribution of homozygote segments in 31 horse autosomes. Figure S3: The distribution of Z(F_st_) and $\log_{2}\left(\frac{{\theta \pi }_{\left(\mathrm{Non}-\mathrm{sport}\right)}}{{\theta \pi }_{\left(\mathrm{Sport}\right)}}\right)$ in 100 kb windows with sliding windows of 50 kb. Figure S4: Biological processes for 49 genes under selective pressures. Table S1: Sequencing and whole-genome mapping information of the animals used in this study. Table S2: Runs of homozygosity results. Table S3: Genomic regions and genes underlying selection pressures.
