# Supplementary figures and images for: Whole-Genome Signatures of Selection in Sport Horses Revealed Selection Footprints Related to Musculoskeletal System Development Processes
Source: Animals (Basel). 2019 Dec 26;10(1):53. doi: 10.3390/ani10010053 (PMC7023322; doi:10.3390/ani10010053)

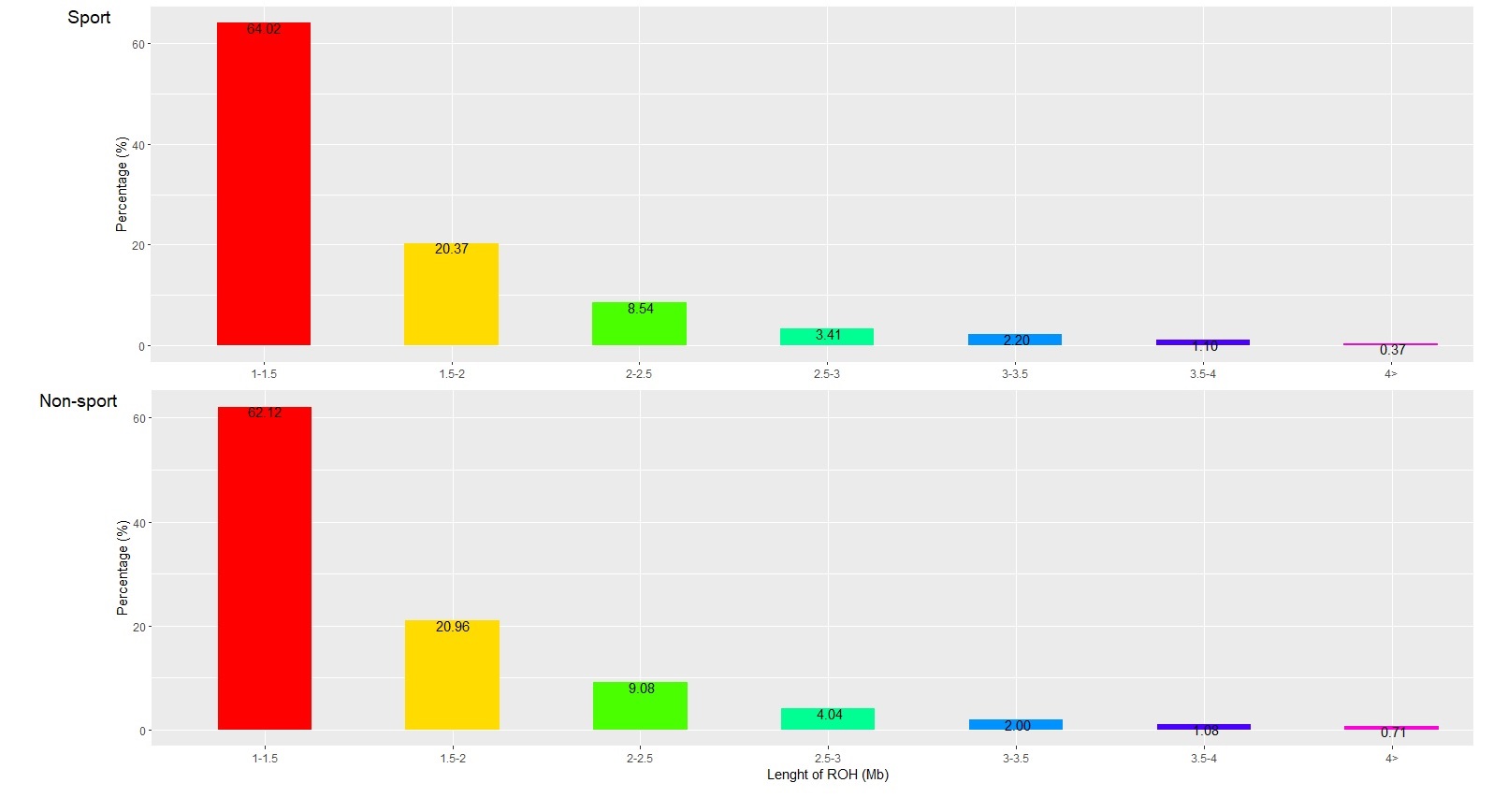

Supplement: Supplementary file 1 [file animals-10-00053-s001.zip › Supplementary/Supplementary Figure 1.jpg]

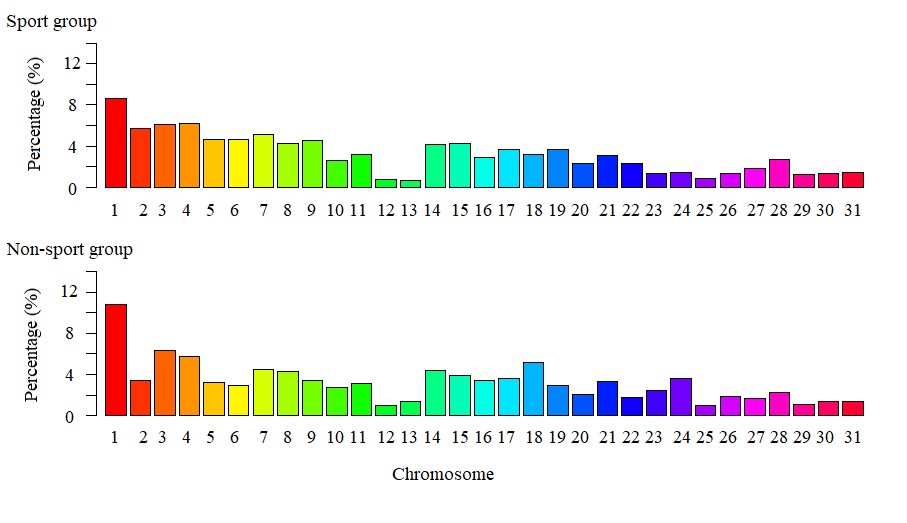

Supplement: Supplementary file 1 [file animals-10-00053-s001.zip › Supplementary/Supplementary Figure 2.jpg]

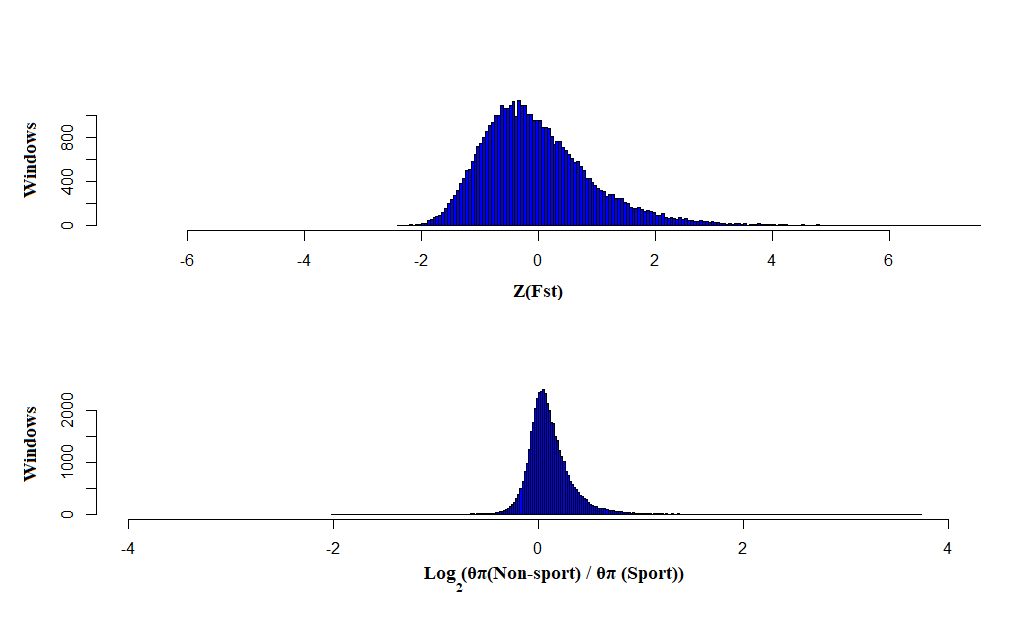

Supplement: Supplementary file 1 [file animals-10-00053-s001.zip › Supplementary/Supplementary Figure 3.tiff]

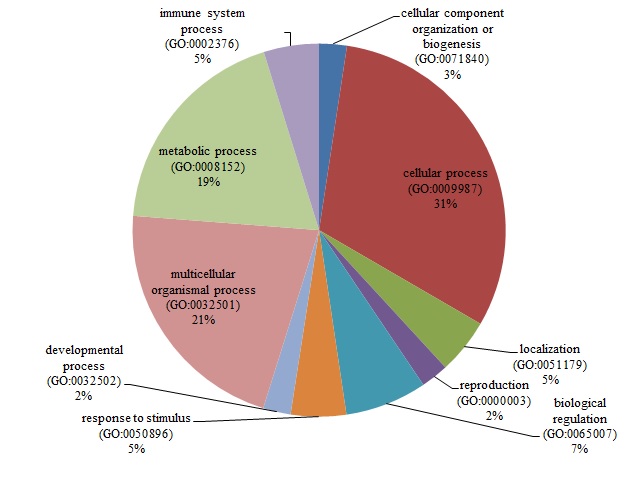

Supplement: Supplementary file 1 [file animals-10-00053-s001.zip › Supplementary/Supplementary Figure 4.jpg]
